# An Ultrasound-Guided Interfascial Injection Approach Versus an Ultrasound-Assisted Nerve Stimulating Approach of Obturator Nerve Block: A Randomized Clinical Trial

**DOI:** 10.7759/cureus.24037

**Published:** 2022-04-11

**Authors:** Brijesh Tiwari, Pranchil Pandey

**Affiliations:** 1 Urology, Shyam Shah Medical College Rewa, Rewa, IND; 2 Anaesthesiology, Shyam Shah Medical College Rewa, Rewa, IND

**Keywords:** bladder tumor, ultrasound-guided, turbt, transurethral resection, obturator nerve block

## Abstract

Background

The majority of bladder cancer patients are elderly and have various comorbidities, increasing the risk of complications following general anesthesia. Spinal anesthesia with a selective obturator nerve block (ONB) is an alternative to general anesthesia for transurethral resection of bladder tumor (TURBT); however, incomplete ONB can cause adductor muscle spasm. The objective of this study was to assess if the ultrasound-guided interfascial injection approach is compatible with the blind nerve stimulating technique for ONB in bladder cancers undergoing TURBT.

Methodology

A total of 50 ONBs were performed for TURBTs under spinal anesthesia and were divided into two groups, that is, ONB with nerve stimulation control group (group RD1) and an experimental ultrasound-guided interfascial injection group (group RD2). During TURBT surgeries, one urology assistant determined obturator reflex grade (I-IV) at 15 minutes after injection completion in both groups.

Results

A success rate of 88% was achieved in group RD1 compared to 76% in group RD2, which was clinically significant. Three cases failed to achieve complete ONB in group RD1, and six cases in group RD2 failed to achieve complete ONB. One case in group RD1 and two cases in group RD2 exhibited grade II obturator re­flex during the surgery.

Conclusions

Ultrasound-guided interfascial injection approach was inferior to the ultrasound-guided nerve stimulating technique for ONB at the inguinal crease; hence, we recommend using both ultrasound and nerve stimulators for ONB.

## Introduction

Transurethral resection of bladder tumor (TURBT) is a primary diagnostic and therapeutic step for bladder tumors; however, direct electrical stimulation of the obturator nerve (ON) dur­ing TURBT can trigger an inadvertent adductor muscle spasm, which can cause severe complications such as bladder perforation [1].

The majority of bladder cancer patients are elderly and have various comorbidities, increasing the risk of complications following general anesthesia. Spinal anesthesia with a selective obturator nerve block (ONB) is an alternative to general anesthesia for TURBT; however, incomplete ONB can cause adductor muscle spasms. Although nerve stimulators have been utilized under ultrasound guidance to improve the performance of ONB, it has recently been reported that ONB can be conducted with similar efficacy using interfascial injection under ultrasound guidance without the use of a nerve stimulator [2].

However, because ON itself is very thin and generally embedded in an inter­muscular septum, it is difficult to be visualized even in ultrasound images and is difficult to be electrically stimulated [2]. Therefore, we were interested in comparing both techniques. The primary objective of this study was to see if an ultrasound-guided interfascial injection is compatible with percutaneous nerve stimulation for TURBT under spinal anesthesia.

## Materials and methods

**Figure 1:**
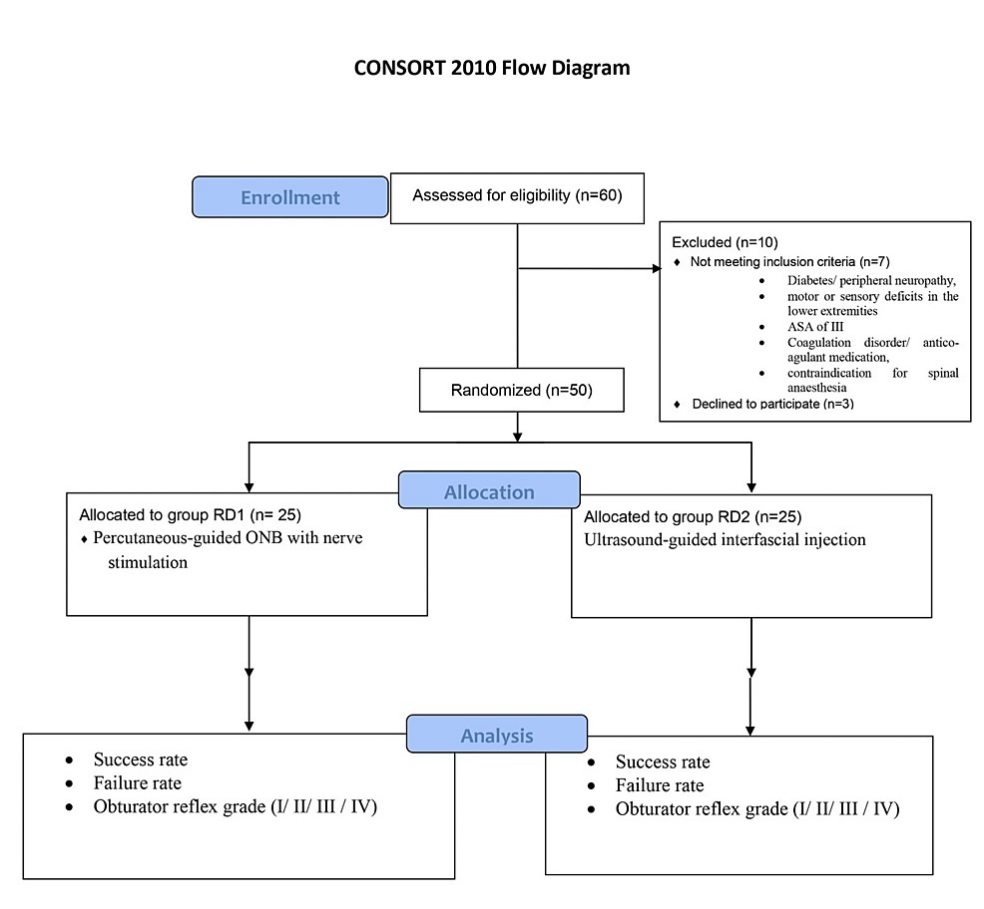
CONSORT 2010 checklist. ONB: obturator nerve block; ASA: American Society of Anesthesiologists

After obtaining approval from the Institutional Ethics Committee (Approval number: IES-SSMC-010) and informed consent from parents, a randomized, prospective, parallel-group study was conducted on 50 patients. All patients underwent spinal anesthesia with ONB for elective TURB for bladder tumors. For a study power of more than 80%, the required sample size was calculated to be approximately 50 patients through a pilot study. The number of groups, standard deviation, and Z power table were used to evaluate it. This study was performed in accordance with the CONSORT 2010 checklist (Figure 1).

The lottery method was used to perform simple randomized sampling. The patients were randomized into two groups of 25 patients each, that is, a percutaneous-guided ONB with nerve stimulation control group (the RD1 group) and an ultrasound-guided inter­fascial injection experimental group (the RD2 group) to receive ONB in the inguinal crease. We excluded patients with diabetes or peripheral neurop­athy, motor or sensory deficits in the lower extremities, ASA of III or greater, a coagulation disorder, antico­agulant medication, known allergy to local anesthetics (LAs), contraindication for spinal anesthesia (infection at the in­jection site, severe scoliosis, or fusion surgery), lack of cooperation, and refusal to participate.

All procedures were conducted in the operating room of the Super Speciality Block, Sanjay Gandhi Memorial Hospital Rewa from September 2020 to September 2021. Routine monitoring was started, and spinal anesthesia with 0.5% bupivacaine 12-15 mg was ad­ministered to achieve anesthesia levels above T10 in all patients. The affected leg was slightly abducted and rotated externally without knee flexion after the patient was placed in a supine position, and the inguinal region was prepped with povidone-iodine solution. A sterile plastic cover and gel were attached to a 10-MHz linear probe, which was positioned parallel to the inguinal crease at 90 degrees to the skin. The inguinal region was examined laterally from the femoral vein until the pectineus muscle was located medially at the inguinal crease with the adductor longus, adductor brevis, and adductor magnus. In the RD1 group, a 22-gauge, 120-mm stimulating needle (Stimuplex insulated needle; D Plus B. Braun, Melsungen, Germany) was advanced in a lateral to medial direction with a nerve stimulator (Stimuplex HNS12; B. Braun, Melsungen, Germany) to position the needle tip at the junction of the adductor longus, adductor brevis, and pectineus muscles within the fascia for an anterior branch. After switching on the nerve stimulator, 10 mL of LA (1.5% lidocaine + epi 1:200,000) was slowly injected into the muscle interface after negative aspiration if adductor muscle twitching was noted even at 0.3 mA.

Without using a nerve stimulator, the same amount of LA was injected at the anterior and posterior branch sites beneath the fascia in the RD2 group. The needle was advanced by the same anesthesiologist five minutes after the major branches were blocked to look for any twitching. We were able to determine that the adductor muscle twitching had stopped in both groups after the LA injection. All of the blocks were performed by a single anesthesiologist with an experience of over 60 ONBs.

According to the study design, the primary outcome was the failure rate of ONB confirmed with the nerve stimulator only. The failure rate in the RD1 group was assumed to be zero in all cases because the twitch of the adductor muscles was confirmed to be absent when the LA was injected. The performer in the RD2 group stopped the first injection using simply the anatomical landmark on the ultrasound picture, then blocked the second injection with a nerve stimulator to assess the twitches on the initial injection site and the failure rate.

The extent of the adductor motor block, as determined by obturator reflex grade, was the secondary outcome. Following ONB, one urology assistant who was not aware of the group allocations entered the operating room, and patients were placed in the lithotomy position. A monopolar resectoscope and irrigation with a glycine solution were used to begin endoscopic resection of the tumor. The surgery was performed by the same surgeon. We asked a urology assistant to perform obturator reflex grading on both groups 15 minutes after injection, as described by Lee et al. [3]: Gr I, no movement or palpable muscle twitching; Gr II, palpable muscle twitching without movement; Gr III, slight movement of the thigh not interfering with the surgical procedure; and Gr IV, vigorous movement interfering with the surgical procedure.

Results are presented as mean standard deviations. The SPSS software version 12 (SPSS Inc., Chicago, IL, USA) was used to conduct the statistical analysis. For categorical data, the chi-square test or Fisher’s exact test were employed. For continuous data, the Student’s unpaired t-test was utilized. P-values of less than 0.05. were considered statistically significant.

## Results

Mean age and sex were comparable between both groups (Table 1). The number of skin punctures to ONB was one for all 50 blocks. The mean duration of surgery in both groups was comparable (Table 2). A success rate of 88% was achieved in group RD1 compared to 76% in group RD2. Three cases failed to achieve complete ONB in group RD1, whereas six cases in group RD2 failed to achieve complete ONB. One case in group RD1 and two cases in group RD2 exhibited grade II obturator re­flex during the surgery (Table 3). No case in either group required general anesthesia to complete the surgery. No neurologic, vascular, or infection-re­lated complications were seen using two different techniques of ONB.

**Table 1:** Demographic data. SD: standard deviation; ASA: American Society of Anesthesiologists

Variables	RD1 (N = 25)	RD2 (N = 25)	P-value
Age, year; mean ± SD	65 ± 7	67 ± 8	0.453
Sex (male/female); N	20/5	21/4	0.654
ASA class (I/II); N	7/18	8/17	0.213

**Table 2:** Obturator nerve block data (intraoperative).

Variables	RD1 (N = 25)	RD2 (N = 25)	P-value	
Duration of surgery (minutes); mean ± SD	60.6 ± 11.2	55.9 ± 17.3	0.564	
Dose of 0.5% bupivacaine (mg); mean ± SD	12.4 ± 1.6	13.1 ± 1.2	0.447	
Spinal level (T10/T8/T6/T4); N	1/4/11/9	3/3/14/5	0.342	
Side (right/left)	11/14	13/12	0.436	

**Table 3:** Obturator nerve block outcomes.

Variables	RD1 (N = 25)	RD2 (N = 25)	P-value	
Success rate; N (percentage)	22 (88%)	19 (76%)	0.267	
Failure rate; N (percentage)	3 (12%)	6 (24%)	0.234	
Obturator reflex grade (I/II/III/IV); N	24/1/0/0	23/2/0/0		

## Discussion

The ultrasound-guided interfascial injection approach and the percutaneous nerve stimulating technique for ONB at the inguinal crease are less efficacious compared to using both ultrasound and nerve stimulators. In the RD2 group, the block was not achieved on six occasions. In this investigation, we used fascia over an ON because ON is tiny and difficult to photograph [1,2]. We confirmed that LA diffused along the adjacent interfascial layers in the RD2 group, rather than remaining stationary on the injection site. Initially, we assumed that the RD2 and RD1 groups would be comparable; however, in certain cases, twitching points outside the area of LA spread were identified. Despite a correct electrical endpoint, incomplete ONB is known to be caused by insufficient LA diffusion [2].

Because several muscle layers are close to the ON pathway, which is interwoven and complicated, minor tilting of the transducer can result in the pathway being missed [4]. When the probe was held perpendicular to the skin, for example, there was no twitching, but twitching occurred when the probe was tilted 10° cranially. The dynamics of nerve position were studied by Saranteas et al., who discovered that probe angulations can modify nerve location within the anatomic line [4]. Because large transducer angles make ONB technically difficult and increase the likelihood of significant complications, we authorized 10-20° angulation of the probe.

Interfascial procedures have been found to have high success rates [2]. The anterior and posterior divisions of ON have various branching patterns that are extensively distributed across the adductor muscles, and intrafascial injection is a volumetric approach that relies on the diffusion of the injected medication [4,5]. According to the authors of a study demonstrating the compatibility of the interfascial injection technique on the ONB, utilizing a nerve stimulator improved the precision of blocking the posterior branch [2]. We used the nerve stimulator again to look for unsuccessful cases in the RD2 group, and more LA was injected for the patients’ safety. Simply stimulating the bladder walls with electric resectoscopes to evaluate the block quality can trigger a severe contraction of the adductor muscles and a bladder perforation. Consequently, we employed a low-level stimulant current (0.3-0.5 mA) to ensure that the needle tip was positioned as close to the nerve as possible and for safety reasons [6].

Even though total ONB was achieved in this trial, we were unable to completely suppress the obturator reflex. Because of the severity of bladder perforation, we are often concerned about even little muscle contractions. However, in practice, even total ONB does not guarantee complete adductor motor block because innervations from the femoral and sacral plexuses also contribute to adductor motor strength. There are no standard definitions for complete ONB, and evaluating ONB in clinical contexts is time-consuming and complicated [2,4].

A major limitation of our study is the limited sample size, which makes it difficult to determine uncommon or infrequent incidents. Even though we recruited 50 patients, the primary outcome was underpowered in the final analysis. One of the disadvantages of this study is that it cannot be classified as a double-blind study. Even though urology assistants who evaluated obturator reflex were unaware of the group assignments because they entered the operating room after ONB, the performers were not blinded to the group assignments (one injection for the RD1 group or two injections for the RD2 group).

## Conclusions

Findings of the current study strongly support that the ultrasound-guided interfascial injection approach was less efficacious and had more block failures compared to the ultrasound-guided nerve stimulating technique for ONB at the inguinal crease. Hence, we recommend using both ultrasound and nerve stimulators for ONB. Furthermore, we want to stress that a successful ONB does not imply an absolute adductor motor block.
